# Mfn2 Ablation in the Adult Mouse Hippocampus and Cortex Causes Neuronal Death

**DOI:** 10.3390/cells9010116

**Published:** 2020-01-03

**Authors:** Song Han, Priya Nandy, Quillan Austria, Sandra L. Siedlak, Sandy Torres, Hisashi Fujioka, Wenzhang Wang, Xiongwei Zhu

**Affiliations:** 1 Department of Pathology, Case Western Reserve University, Cleveland, OH 44106, USA; 2 Department of Pathophysiology, School of Basic Medical Sciences, Wuhan University, Wuhan 430072, China; 3 Electron Microscopy Core Facility, Case Western Reserve University, Cleveland, OH 44106, USA

**Keywords:** mitofusin-2, Alzheimer’s disease, neurodegeneration, mitochondrial fragmentation, oxidative stress, mitochondrial ultrastructural damage, aberrant cell-cycle event, apoptosis

## Abstract

It is believed that mitochondrial fragmentation cause mitochondrial dysfunction and neuronal deficits in Alzheimer’s disease. We recently reported that constitutive knockout of the mitochondria fusion protein mitofusin2 (Mfn2) in the mouse brain causes mitochondrial fragmentation and neurodegeneration in the hippocampus and cortex. Here, we utilize an inducible mouse model to knock out Mfn2 (Mfn2 iKO) in adult mouse hippocampal and cortical neurons to avoid complications due to developmental changes. Electron microscopy shows the mitochondria become swollen with disorganized and degenerated cristae, accompanied by increased oxidative damage 8 weeks after induction, yet the neurons appear normal at the light level. At later timepoints, increased astrocyte and microglia activation appear and nuclei become shrunken and pyknotic. Apoptosis (Terminal deoxynucleotidyl transferase dUTP nick end labeling, TUNEL) begins to occur at 9 weeks, and by 12 weeks, most hippocampal neurons are degenerated, confirmed by loss of NeuN. Prior to the loss of NeuN, aberrant cell-cycle events as marked by proliferating cell nuclear antigen (PCNA) and pHistone3 were evident in some Mfn2 iKO neurons but do not colocalize with TUNEL signals. Thus, this study demonstrated that Mfn2 ablation and mitochondrial fragmentation in adult neurons cause neurodegeneration through oxidative stress and neuroinflammation in vivo via both apoptosis and aberrant cell-cycle-event-dependent cell death pathways.

## 1. Introduction

Alzheimer’s disease (AD) is a neurodegenerative disease characterized by progressive neuronal loss and the presence of hallmark neuropathology of neurofibrillary tangles and amyloid plaques, especially in the hippocampus and cortex [1]. Defective glucose utilization and energy metabolism is one of the best-documented abnormalities in AD, implicating an essential role of mitochondrial dysfunction in the pathogenesis of AD [2]. Indeed, mitochondrial dysfunction and oxidative stress have been found as an early and prominent feature in AD patients and AD animal models [2,3]. However, the underlying mechanism remains elusive. 

Some early studies demonstrated intriguing changes in mitochondrial morphology associated with a swollen and damaged appearance and severe loss of inner cristae structure in the cortical neurons in the AD brain [4,5], although the significance remained unclear until major efforts were devoted in the study of mitochondrial dynamics in neurodegeneration [6]. Mitochondria are constantly undergoing fission and fusion processes, which allow for the transfer of molecules to maintain mitochondria homeostasis, including mitochondrial segregation, transport, and degradation [7,8]. The processes of fission and fusion are regulated by a group of GTPases. Mitochondrial fission is mediated by dynamin-like protein 1 (DLP1), assisted by mitochondrial outer membrane protein factors including fission 1 (Fis1) and mitochondrial fission factor (Mff), while fusion is mediated by Mitofusin 1 and 2 (Mfn1 and Mfn2) on the mitochondrial outer membrane and optic atrophy protein 1 (OPA1) on the mitochondrial inner membrane [7,8]. Mutations in genes encoding many of these proteins cause neurological disorders in humans [9]. For example, Mfn2 deficiency causes Charcot–Marie–Tooth (CMT) disease type 2A in 20–40% of CMT cases [10,11], while OPA1 mutations cause dominant optic atrophy (DOA) [12]. The fission-related protein DLP1 is reported to be associated with refractory epilepsy and neonatal lethality [13], while Mff is linked to developmental delay with neuromuscular dysfunction [14]. Interestingly, studies from multiple groups demonstrated fragmented mitochondria in cell models and animal models of AD, as well as in the pyramidal neurons in the brains of human AD patients [15,16,17,18,19,20,21,22]. Consistently, decreased mitochondria fusion proteins including OPA1, Mfn1, and Mfn2, along with increased fission proteins such as Fis1 and DLP1, are found in the AD brain and models of AD, which suggests that impaired fusion and/or enhanced fission is likely involved [21,22,23].

Excessive mitochondrial fission and its deleterious effect were consistently demonstrated in cell models of AD in vitro, where overexpression of APP or Aβ treatment induces profound mitochondrial fragmentation, ultrastructural deficits, and altered distribution in neuronal cultures [15,16,17,18,21,22], which are likely causally involved in Aβ-induced synaptic abnormalities in hippocampal neurons. Our prior studies demonstrated that abnormal mitochondrial dynamics, caused by conditional genetic ablation of Mfn2, cause mitochondrial dysfunction and neurodegeneration in AD-affected brain areas, which supports a causal role of abnormal mitochondrial dynamics in mitochondrial dysfunction and neurodegeneration in vivo [24]. However, since Mfn2 is knocked out at as early as 2 or 3 weeks of age [25,26,27], a potential contribution of abnormal developmental changes to our observations may not be completely ruled out. To better understand the contribution of abnormal mitochondrial dynamics in age-related neurodegeneration disorders such as AD, investigation of a Mfn2 knockout mouse model in adult neurons will be more convincing. Therefore, in this study we have utilized the CaMKCreERT2 mouse model to induce ablation of Mfn2 in forebrain neurons in adult mice and studied the effect of Mfn2 ablation in adult neurons.

## 2. Materials and Methods

### 2.1. Mouse Model

To generate the Mfn2KO mouse, CaMKII^CreERT2^ mice [28] were crossed with Mfn2 floxed mice. Mice were housed and maintained at the Case Western Reserve University Animal Resource Center under approved IACUC protocol #2015-0068 (Approval data: 29 May 2015). Upon weaning, ear punches were taken to both identify and genotype each mouse. To knockout Mfn2, Cre expression was induced in CAMKII-expressing neurons in adult mice by injection with 100 µL of tamoxifen (10 mg/mL in a 10:1 mixture of peanut oil:ethanol) ip twice daily for 5 days. Mice were maintained for up to 10 months (40 weeks) post-induction, and no change in feeding, weight, or lethality was noted for this model. At 4 weeks post-induction, recombination of the floxed alleles was examined in the dissected cortex, hippocampus, and cerebellum of double transgenic mice by PCR using Mfn2-F (GAAGTAGGCAGTCTCCATCG) and Mfn2 ex-R (CCCAAGAAGAGCATGTGTGC) primers.

At least 3 mice from each experimental group were euthanized at 8, 9, 12, 16, and 40 weeks post-induction, and brain tissue was collected. One half of the brain was fixed in buffered formalin and embedded in paraffin for histochemistry and immunocytochemistry. The other half was dissected, and the cortex, hippocampus, and cerebellum were frozen at −80 °C for biochemical analysis. For some mice, one half-brain was fixed for electron microscopy (as per the method outlined below). 

Control non-induced mice included WT (Wild type), ERT+/Mfn2^+/+^ or ERT+/Mfn2^+/−^ mice injected with the vehicle (peanut oil) or no injection, and Mfn2^+/+^ or Mfn2^+/−^ mice injected with 100 µL of tamoxifen (10 mg/mL in a 10:1 mixture of peanut oil:ethanol) ip twice daily for 5 days. Mice were matched for gender, age of induction, and induction duration. Control mice were maintained for up to 40 weeks after injection and brain tissue cut sagittally; one half was stored at −80 °C, and the other half was fixed in buffered formalin and embedded in paraffin. These mice were sacrificed at comparable ages for all groups (control mice: 39.4 ± 2.4 weeks; Mfn2 iKO mice: 37.5 ± 2.8 weeks) except for the group with a 40-week induction (control mice: 75 ± 0.9 weeks; Mfn2 iKO mice: 79 ± 2.3 weeks).

### 2.2. Immunoctyochemistry

Following fixation in formalin, sagittal brain tissues were embedded in paraffin and 6 micron thick sections were prepared. Tissue sections were deparaffinized in two changes of xylene and rehydrated through changes of 100% ethanol, 95% ethanol, 70% ethanol, and 50% ethanol to Tris buffered saline (TBS, 50 mM Tris, 150 mM NaCl, pH = 7.6). For most experiments, antigen retrieval was performed using citrate buffer and pressure cooking (Biocare, Pacheo, CA, USA). After thoroughly rinsing, sections were blocked with 10% normal goat serum (NGS) in TBS for 30 min and primary antibodies applied and incubated overnight at 4 °C. After rinsing in 1% NGS and a 10 min step in 10% NGS, secondary antibodies were applied for 30 min, followed by another rinsing step and 1 h incubation in mouse or rabbit peroxidase anti-peroxidase (PAP) complex. Sections were rinsed 3 times in Tris buffer (pH = 7.6) and developed with a 3′,3′-diaminobenzidine (DAB) chromogen kit (ENZO, Farmingdale, NY, USA). Slides were dehydrated and coverslipped using Permount. For some experiments, double-labeling fluorescent microscopy was used. Sections were prepared as above, and primary antibodies were incubated together overnight. After rinsing and blocking in 10% NGS, Alexafluor 488 and 568 conjugated secondary antibodies (Invitrogen, Carlsbad, CA, USA) were applied at 1/300 dilution for 1 h at room temperature. After rinsing thoroughly in phosphate-buffered saline, sections were stained with 4′,6-diamidino-2-phenylindole (DAPI) and coverslipped using Fluoromount-G (Southern Biotech, Birmingham, AL, USA).

Antibodies used include mouse monoclonals for detecting NeuN (Millipore, Danvers, MA, USA), 4-hydroxy-2-nonenal (HNE) (Jaica, Carlsbad, CA, USA), Mfn2 (Santa Cruz, Dallas, TX, USA), mitochondria oxidative phosphorylation (OXPHOS) (Abcam, Cambridge, MA, USA), glial fibrillary acidic protein (Invitrogen), iba1 (Invitrogen), and heme oxygenase-1 (HO1, Enzo, Farmingdale, NY, USA). Cell cycle markers included rabbit phosphor-histone H3-Ser10 (pH3-Ser10, Cell Signaling, Danvers, MA, USA) and mouse and rabbit proliferating cell nuclear antigen (PCNA, Cell Signal and Abcam). A TUNEL assay (Roche, Indianapolis, IN, USA) was performed according to the manufacturer’s instructions. A DNP assay was performed as previously described [29] and briefly, 2-nitro-diphenylhydrazine was incubated on the tissue sections for 15 min at 37 °C. After rinsing thoroughly in acetic acid and then in TBS, rabbit anti-DNP was applied (Dako, 1/10,000, Santa Clara, CA, USA) using a routine immunostaining method. 

For light microscopy, all images were taken with a Zeiss Axiophot and Axiocam with Axiovision software Rel.4.8. Images of the hippocampus region of neuronal nuclei (NeuN)-, glial fibrillary acidic protein (GFAP)-, and iba1-stained sections were used to quantify the relative levels as % area stained, using Axiovision software. The immunostained structures were measured as percent area covered in the entire hippocampus region. This quantifies the neuronal shrinkage and cell loss for NeuN and the extent of glial cell activation for GFAP and iba1. For densitometric analysis of DNP, 4-hydroxynonenal (HNE), and HO-1 staining, hippocampal images were obtained and the mean densitometric values relative to blank tissue areas for each section were obtained with Axiovision software. One-way ANOVA with Tukey’s post hoc analysis and t-tests were used to determine significance. Fluorescent images were obtained on a Zeiss Axiovert microscope with Axiovision software.

### 2.3. Western Blot

Brain cortex regions were collected and stored at −80 °C for western blot analysis. To prepare lysates for western blot analysis, frozen samples were homogenized on ice with a Dounce homogenizer using lysis buffer (Cell Signaling, Danvers, MA, USA) containing protease and phosphatase inhibitors (Roche). A bicinchoninic acid assay (BCA) assay was used to determine protein concentration. Per lane, 15 µg of protein was separated on 10% SDS-PAGE gel and transferred to immobilon membrane. After blocking in 10% dry milk in TBS containing 1% Tween20, primary antibodies were incubated overnight with shaking at 4 °C. In addition to the antibodies listed above, rabbit polyclonal to Glyceraldehyde 3-phosphate dehydrogenase (GAPDH) (Cell Signaling, Danvers, MA, USA) was used for loading control. Thorough rinsing in TBST (TBS with 0.1% Tween-20) and secondary antibodies conjugated to Horseradish peroxidase (HRP) were incubated for 1 h at room temperature on a shaker. Finally, after rinsing 5 times in TBST, blots were developed with ECL reagent (Santa Cruz, Dallas, TX, USA or Millipore, Danvers, MA, USA) and bands detected with Amersham imager. Target bands were quantified with QuantityOne (Biorad, Hercules, CA, USA). The student’s *t*-test was used for statistical analysis.

### 2.4. Electron Microscopy

For electron microscopy analysis, induced mice maintained for 8 weeks and 40 weeks post-induction and control mice consisting of no ERT gene with tamoxifen (one each at age 12 weeks and 40 weeks) or with ERT gene with vehicle injection (40 weeks) were perfused with saline, and their brains were immediately dissected and placed into fixative, as previously described. Using a razor blade, thin sagittal slices were prepared (approximately 0.5 mm thick) containing the entire hippocampal formation and cortex. Small square areas containing the CA1 region and the cortex region located just above the hippocampus were carefully dissected and embedded into Epon. Semi-thin sections were stained with toluidine blue to identify the correct neuronal layers for imaging. Electron microscopy images were collected of 6–8 representative neurons from each mouse. 

Using Image J, all mitochondria from those neurons were measured for length, width, and area (size) and the mitochondria aspect ratio calculated. The student’s t-test was used for statistical analysis.

## 3. Results

### 3.1. Recombination Results in Decreased Mfn2 Levels

**Figure 1:**
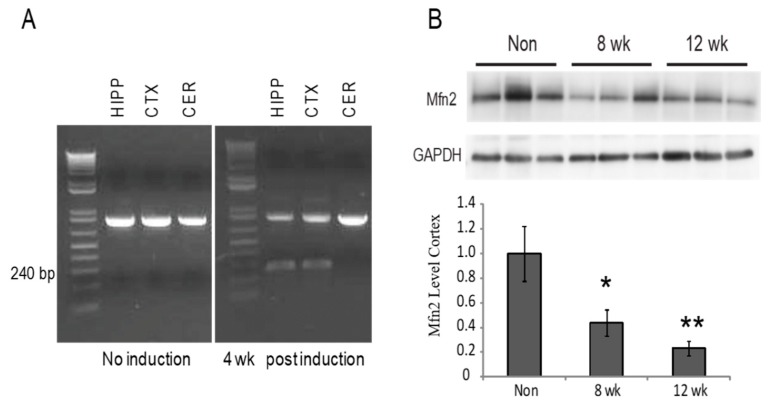
Decreased Mfn2 protein levels following recombination. PCR confirms the recombination product in the brains of transgenic mice, and 4 weeks after induction with tamoxifen, the hippocampus and cortex also had the excised band at 240 bp, while the cerebellum did not have the excised band (**A**). Western blot analysis of cortical lysates found significant loss of Mfn2 protein in the 8 weeks and 12 weeks post-induction Mfn2 iKO mice (**B**) * *p* < 0.05; ** *p* < 0.01.

Significant Mfn2 reduction was consistently reported in the brain of AD patients and amyloid precursor protein (APP) transgenic mouse models [21,22]. To better understand the role of Mfn2 reduction in the brain areas affected in AD in adult mice, we bred the homozygous Mfn2^fl/fl^ mice with CaMKII^CreERT2^ mice to generate Mfn2^fl/fl/^CAMKII^CreER^ mice, which express Cre-recombinase driven by the CaMKII promoter in the forebrain neurons after treatment with tamoxifen. PCR analysis showed the presence of the floxed Mfn2 transgene product in the hippocampus, cortex, and cerebellum in all non-induced mice examined. Brain tissue collected 4 weeks following induction with tamoxifen (Mfn2 iKO mice), showed an additional 240 kb band representing the excised band in the hippocampus and cortex, and no excision band in the cerebellum, in agreement with the published report on this model (Figure 1A) [28]. A weak excision band was seen in the hippocampus in only one mouse that was not induced (data not shown), suggesting little Cre recombination leakage may occur without tamoxifen induction. Western blot analysis of brain lysates showed significant reduction in the protein expression of Mfn2 in the Mfn2 iKO mice 8 weeks and 12 weeks post-induction compared to control mice (i.e., mice lacking either the ERT or Mfn2 floxed gene injected with tamoxifen, or the Mfn2^fl/fl^CAMKII^CreER^ oil-injected control mice) (Figure 1B).

### 3.2. Mitochondria Distribution and Complex Proteins are Decreased in Neurons

**Figure 2:**
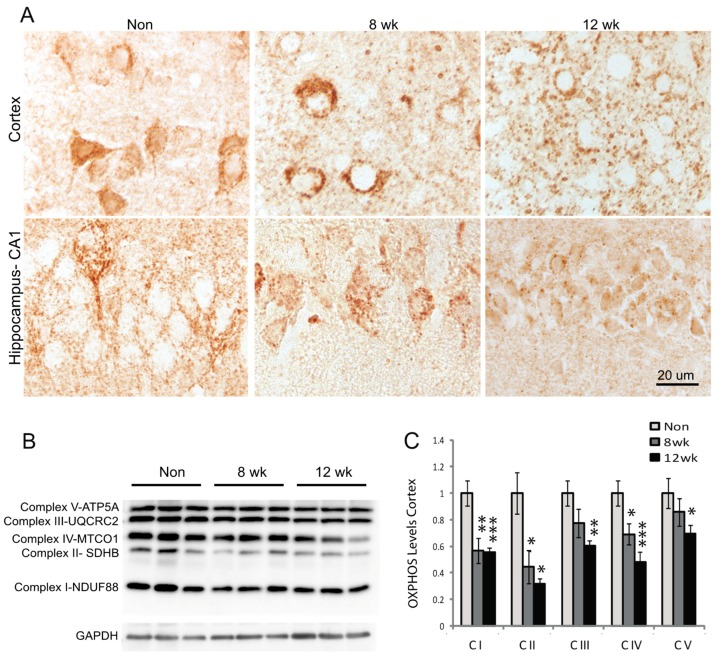
Loss of Mfn2 results in mitochondria abnormalities. Mitochondria immunostained using monoclonal OXPHOS antibody were consistently found throughout the soma and neuronal processes in hippocampal and cortical neurons in non-induced control mice (**A**). At 8 weeks post-induction, mitochondria were found clustered in the soma and not the processes, and some larger round structures, presumably swollen mitochondria, were found in the cortex (**A**). By 12 weeks post-induction, more, larger round structures approximately 0.5–2 microns in diameter were present throughout the cortex, and the hippocampal mitochondria staining was greatly reduced (**A**). By western blot analysis, mitochondria complexes I–V were all found decreased by 12 weeks post-induction (**B**). Quantification, relative to GAPDH as a loading control, determined that even at 8 weeks, complexes I, II, and IV were significantly reduced (**C**). * *p* < 0.05; ** *p* < 0.01; *** *p* < 0.001.

To examine how the reduction of Mfn2 levels in neurons affects mitochondria, sagittal tissue sections were immunostained for mitochondria with monoclonal antibody against mitochondria complex proteins. In all non-induced mice including those lacking either the ERT or Mfn2 floxed gene, or the Mfn2^fl/fl^CAMKII^CreER^ oil-injected control mice, mitochondria in cortical and hippocampal CA1 neurons were visualized throughout the cytoplasm and in many neuronal processes (Figure 2A). The size of the cell bodies and unstained nuclear regions helped identify these cells as neurons. In contrast, by 8 weeks post-induction in the Mfn2 iKO mice, mitochondria were only found in the neuronal soma and few to no processes were immunolabeled, and at 12 weeks post-induction even fewer neuronal cell bodies were clearly identified. Most apparent, however, was the increased number of larger, round structures approximately 1–2 microns in diameter throughout the cortex (Figure 2A). These structures, immunolabeled with monoclonal antibody to mitochondria complex proteins, were present at 8 weeks and even more abundant at 12 weeks post-induction and were not always readily associated with a neuronal cell body. By western blot analysis, mitochondria complexes I, II, and IV were all decreased by 8 weeks post-induction, and complexes III and V were also decreased by 12 weeks post-induction compared to non-induced control mice (Figure 2B,C).

### 3.3. Mitochondria Ultrastructural Changes Apparent at 8 Weeks Post-Induction

**Figure 3:**
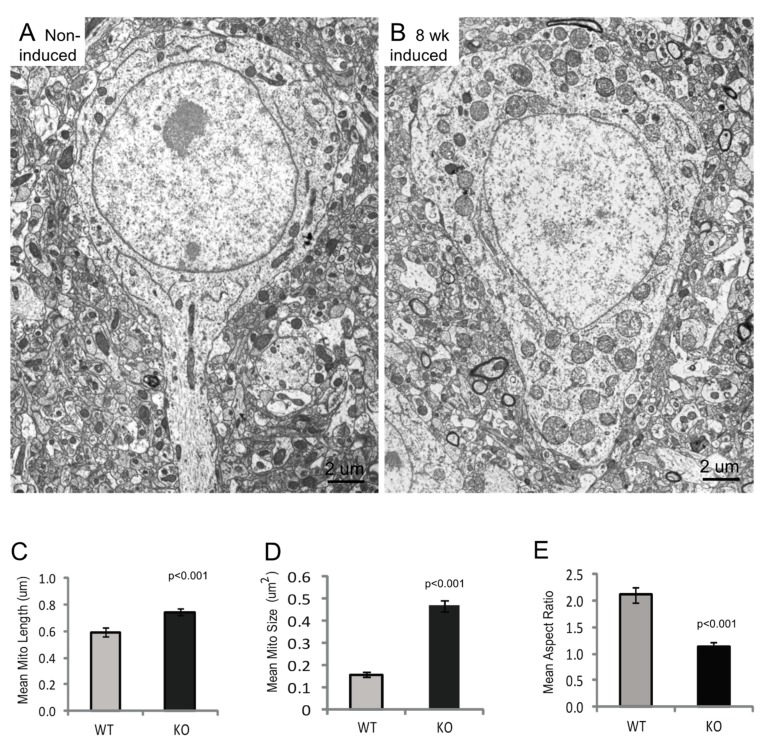
Loss of Mfn2 results in mitochondria ultrastructural abnormalities. Electron micrographs from 8 weeks post-induction mice show that the mitochondria in Mfn2 iKO neurons appear swollen and enlarged (**B**) compared with neurons from non-induced mice (**A**). Quantification of all mitochondria in 8 neurons from at 8 weeks post-induction and 14 neurons from non-induced control neurons found that the mitochondria were approximately 25% longer (**C**) but about threefold larger overall (**D**). Measuring the aspect ratios (the ratio of length/width as a measure of roundness) determined that the mitochondria from normal mice had many typical long and thin mitochondria, though all mitochondria in the Mfn2KO mice were very round, with a mean aspect ratio near 1 (**E**). WT (Wild type), KO (Mfn2 iKO).

To examine how loss of Mfn2 affects mitochondria structure, half-brain samples from Mfn2 iKO mice 8 weeks post-induction and from age-matched non-induced control mice were fixed for electron microscopic analysis. For non-induced control mice, this included the examination of neurons from an oil-injected Mfn2^fl/fl/CAMKCreER^ mouse and from a tamoxifen-injected mouse lacking the ERT+. No qualitative or quantitative mitochondrial differences were noted between the various control conditions, where many thin, tubular mitochondria with intact cristae were observed in neurons (Figure 3A). However, in the Mfn2 iKO mice at 8 weeks post-induction, hippocampal neurons and many cortical neurons exhibited mitochondria swelling and abnormalities in cristae structure (Figure 3A,B). Indeed, many of these swollen and rounded individual mitochondria were between 1 and 2 microns in diameter and likely represented the large and round immunostained structures seen at the light level and shown in Figure 2A. Quantification of mitochondria parameters found that the Mfn2 iKO neuronal mitochondria were approximately 25% longer, on average, compared to those in non-induced mice (Figure 3C), likely reflecting their swollen morphology; indeed, the mean mitochondria size was almost threefold larger in the Mfn2 iKO neurons (Figure 3D). Looking at the mitochondria aspect ratio, defined as length/width, normal mouse neuronal mitochondria had a varied distribution, with many long and thin mitochondria, and the aspect ratios had a positive correlation; however, the mitochondria in Mfn2 iKO neurons all had aspect ratios near 1, representing their swollen, rounded appearance (Figure 3E). No long, thin mitochondria remained in the Mfn2 iKO neurons.

**Figure 4:**
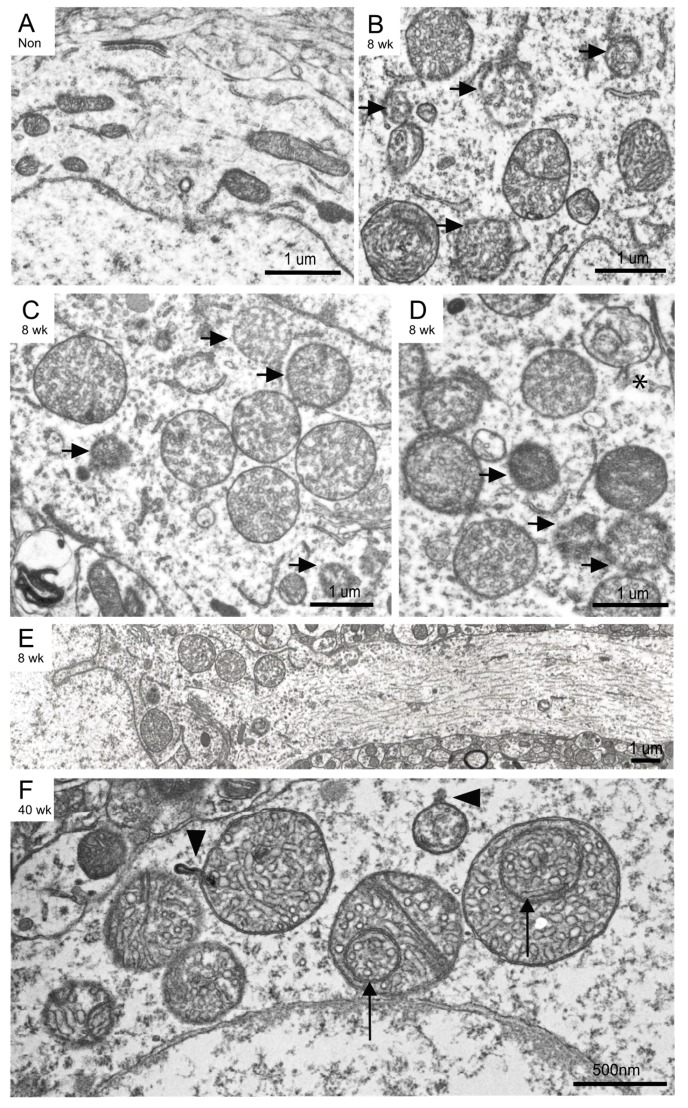
Other ultrastructural defects included cristae and membrane degeneration. Mitochondria abnormalities in the 8 weeks post-induction Mfn2KO neurons included degenerating outer membranes (arrows) and small circular cristae (**B**–**D**), which are compared with normal tubular mitochondria with intact cristae from a non-induced mouse with the same genotype shown at the same magnification (**A**). Some mitochondria have a ruptured outer membrane ((**D**), *). In the Mfn2KO mice neurons, mitochondria were often clustered only in the soma, with few to none present along the processes (**E**), resembling the immunostaining pattern shown in Figure 2. At 40 weeks post-induction, the remaining neurons had mitochondria with internal circular cristae and compartmentalization ((**F**), arrows), and some mitochondria had protrusions ((**F**), arrowheads).

At higher magnification, mitochondria in the non-induced mouse appeared normal with intact double membranes and well-defined cristae often spanning the width of the mitochondria (Figure 4A). Examination of the Mfn2 iKO degenerating mitochondria at higher magnification found that most mitochondria lost the normal cristae structure. Many of the swollen mitochondria contained numerous small vesicular cristae with no connections to the external compartment, and no cristae were seen in the normal configuration, i.e., never spanning the width of the mitochondria (Figure 4B–D, representing mitochondria from three individual neurons). In addition to the extensive cristae structure abnormalities, many mitochondrial outer membranes appeared to be in various stages of degeneration (Figure 4B–D, arrows). These abnormal outer membranes appeared as diffuse structures, lacking the double membrane configuration, and some remnant mitochondria were often just a cluster of damaged cristae with barely visible outer membranes remaining (Figure 4B). Also occasionally found were ruptured mitochondrial membranes creating an opening and possible loss of mitochondrial contents (Figure 4D, asterisk).

Another feature often seen in the 8 weeks post-induction Mfn2 iKO neurons was the lack of mitochondria extending down the axon, with mitochondria only present in the soma (Figure 4E). This reinforces the finding of mitochondria immunostaining only in cell bodies and not in processes in the 8 weeks post-induction Mfn2 iKO mice, as presented in Figure 2A.

We also examined surviving cortical neurons that remained in 40 weeks post-induction Mfn2 iKO mice. In addition to the cristae and outer membrane abnormalities observed in the neurons in the 8 weeks post-induction Mfn2 iKO samples, many mitochondria had large internal circular structures appearing as mitochondria within mitochondria (Figure 4F, arrows). Mitochondria compartmentalization elements and mitochondria with small protrusions (Figure 4B, arrowheads) were also found.

### 3.4. Neurodegeneration Is Accompanied by Inflammation Response in Mfn2 Iko Mice

**Figure 5:**
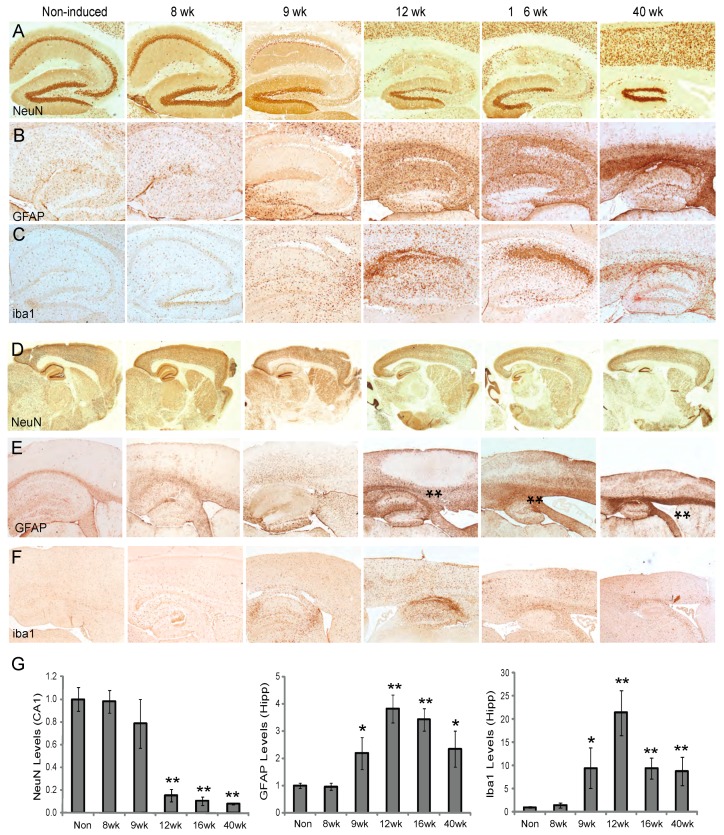
Neurodegeneration and increased inflammatory response in Mfn2 iKO mice. In the hippocampus, neurons did not show evidence of neuronal degeneration until 9 weeks post-induction of Mfn2KO, when all CA1, CA2, CA3, and some dentate gyrus neurons were affected. This hippocampus continues to degenerate and shrink with time (**A**), and NeuN loss significantly decreased starting at 12 weeks post-induction (**G**). Increased activated astrocytes and microglia were apparent at 8 weeks post-induction in the hippocampus (**B**,**C**) and became significantly increased at 9 weeks post-induction (**G**). In the cortex, neuronal degeneration was also accompanied by increased astrocyte and microglial activation (**D**–**F**), and cortical thinning and atrophy was apparent in the later timepoints. Starting at 12 weeks post-induction mice, while some cortical areas were completely covered with GFAP immunoreactivity, other areas remained devoid of astrocytes ((**E**), **). * *p* < 0.05; ** *p* < 0.01.

To determine how ablation of Mfn2 and the resulting mitochondria abnormalities affect neurons in the hippocampus and cortex with age, NeuN immunostaining was performed. In the hippocampus, NeuN immunostaining appeared normal in both the non-induced control mice and Mfn2 iKO mice 8 weeks post-induction. However, by 9 weeks post-induction, there was some loss of NeuN in the CA regions, and a total loss of CA1, CA2, and CA3 neurons was seen by 12 weeks post-induction in the Mfn2 iKO mice (Figure 5A). The hippocampus continued to degenerate at 16 weeks, and at 40 weeks post-induction only the dentate gyrus neurons remained in the Mfn2 iKO mice. The dentate gyrus neurons displayed variable resistance to degeneration among different Mfn2 iKO mice, likely reflecting variability in the induction system. Astrocyte and microglia activation, labeled by antibodies to GFAP and iba, respectively, were increased in the hippocampus starting at 9 weeks and continued to increase at the later timepoints in the Mfn2 iKO mice (Figure 5B,C). Quantification showed the significant loss of NeuN in the CA1 at 12 weeks post-induction, while the hippocampal GFAP and iba1 levels were significantly increased earlier, at 9 weeks post-induction, in the Mfn2 iKO mice (Figure 5G).

Examination of the entire brain found that non-induced control mice and 8 weeks post-induction Mfn2 iKO mice showed no degeneration by NeuN staining, yet starting at 9 weeks post-induction, hippocampal and cortical neuronal loss was seen, and by 16 weeks and 40 weeks post-induction, cortical atrophy was also apparent in the Mfn2 iKO mice (Figure 5D). Brain weight loss was significant starting at 16 weeks post-induction in the Mfn2 iKO mice. Mean brain weight of non-induced control mice was 500.2 ± 16.3 mg, and at 16 weeks post-induction the mean brain weight was 423.3 ± 30.7 mg (*p* < 0.005) in the Mfn2 iKO mice. Increased GFAP and iba1 immunostaining was found in the cortex as well as in the hippocampus starting at 9 weeks post induction in the Mfn2 iKO mice (Figure 5E,F). Also seen in the cortex were many areas devoid of astrocytes in the Mfn2 iKO mice starting at 12 weeks post-induction (Figure 5E, asterisks), which often corresponds with increased microglia activation (Figure 5F) and loss of NeuN (not shown). Microglial activation appeared to reach a peak around 12 weeks post-induction and gradually declined, while astrocyte activation continued to increase until 40 weeks post-induction.

### 3.5. Oxidative Stress and Response

**Figure 6:**
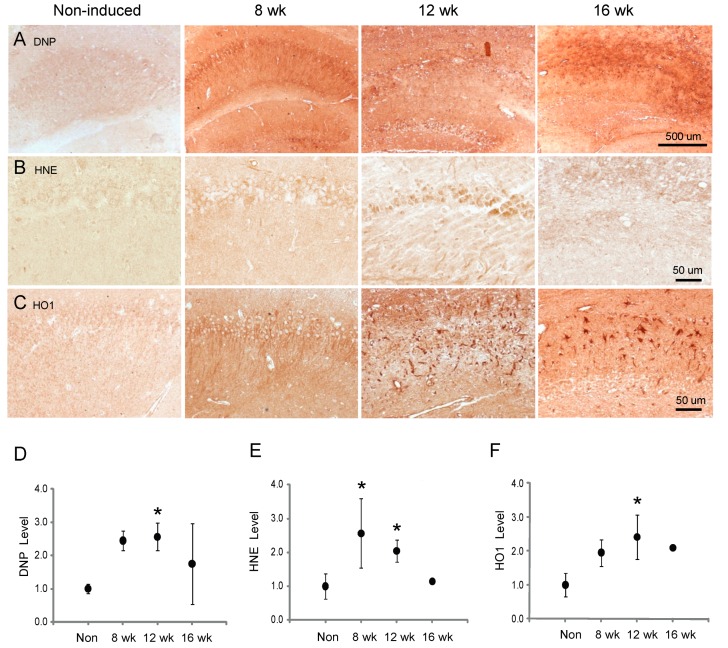
Increased oxidative damage and response is an early feature in this model. As early as 8 weeks post-induction, increased protein oxidation is seen in hippocampal neurons by utilizing the dinitrophenol (DNP) assay for protein carbonyl formation (**A**,**D**). Lipid peroxidation measured by immunohistochemistry for 4-hydroxy-2-nonenal (HNE) is also increased (**B**,**E**). There is a concomitant response of HO-1 induction in both the neurons and glial cells (**C**,**F**). * *p* < 0.05, ANOVA.

The increased oxygen radicals produced following mitochondrial dysfunction often result in oxidative damage. To examine the extent of oxidative damage in the inducible Mfn2 iKO mice, protein carbonyl production was measured using the DNP assay, as well as the levels of lipid peroxidation using an antibody to 4-hydroxynonenal (HNE). Starting at 8 weeks post-induction, there was increased DNP labeling throughout the hippocampus, and at 12 weeks theMfn2 iKO mice had over twofold higher protein carbonyl accumulation than non-induced control mice (*p* < 0.05) (Figure 6A,D). HNE levels in the hippocampus were also significantly elevated in the 8 and 12 week Mfn2 iKO mice, specifically in the neurons (Figure 6B,E; *p* < 0.05). Concomitant to the oxidative insult seen in the hippocampus, there is also an increased oxidative stress response in the Mfn2 iKO mice, visualized by HO-1 levels, a protein induced following oxidative stress (Figure 6C,F; *p* < 0.05 at 12 weeks post-induction). Both neurons and glia demonstrated an increase in HO-1 protein (Figure 6C).

### 3.6. Degenerating Neurons Express Cell Cycle Proteins or Apoptosis

**Figure 7:**
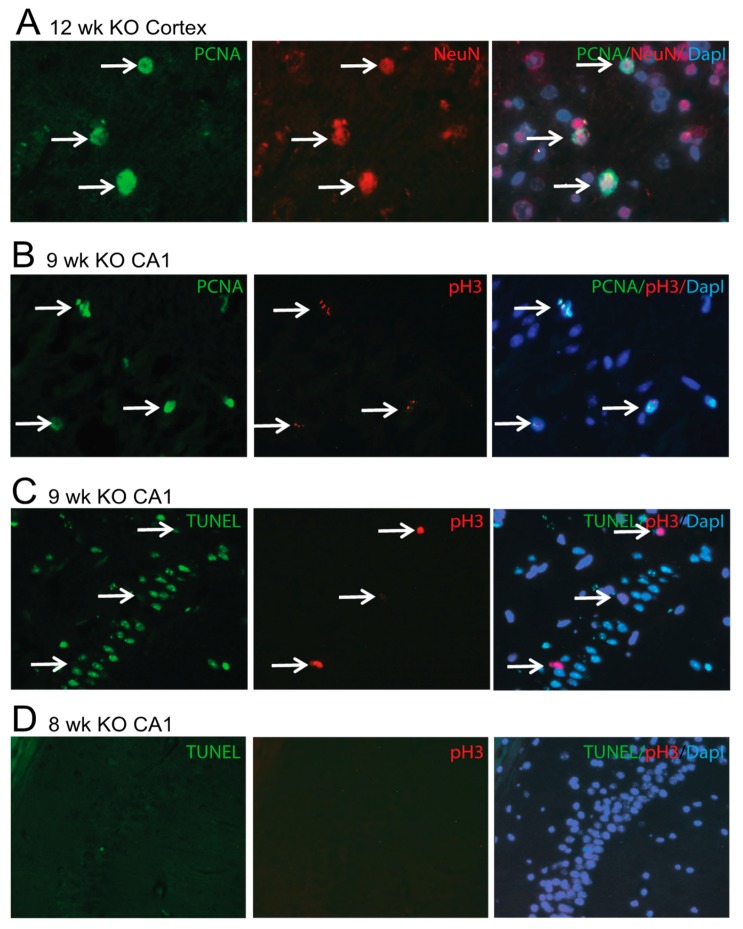
Neurons express cell cycle markers or TUNEL in Mfn2 iKO mice. In the cortex of 12 weeks post-induction Mfn2 iKO mice, some neurons stained with NeuN also had increased levels of the early cell cycle marker proliferating cell nuclear antigen (PCNA) (**A**). In a 9 weeks post-induction mouse, the early and late cell cycle markers, PCNA and pH3-Ser10, were often found colocalized in neurons (**B**). Apoptotic cells stained by TUNEL were present in the CA1 region at 9 weeks post-induction and were never labeled by pH3-Ser10 (**C**). In no 8 weeks post-induction neurons were there any cell cycle markers or TUNEL expressed (**D**).

Cell cycle reactivation has been documented to occur in a variety of neurodegenerative diseases and models [30]. To determine whether cell cycle proteins are expressed in the Mfn2 iKO mice, early and late cell cycle markers were examined. Proliferating cell nuclear antigen (PCNA) is expressed during the G1/S (early) phase through the G2 (late) phase of the cell cycle. In the Mfn2 iKO mice, some neurons in the 9 weeks post-induction hippocampus and 12 weeks post-induction cortex expressed PCNA (Figure 7A, NeuN red; PCNA green). Also examined was the G2/M late cell cycle marker, phosphorylated-Histone H3 Ser10 (pH3-Ser10). Many neuronal nuclei were stained by both PCNA (Figure 7B green) and pH3-Ser10 (Figure 7B, red). Other smaller PCNA-positive nuclei were in microglia, as determined by co-staining with iba1, but were never associated with GFAP-positive astrocytes (not shown). pH3-Ser10 was never found to be localized in microglia (not shown).

While some neurons were seen undergoing cell cycle re-entry, apoptosis was a more prominent finding. In the Mfn2 iKO mice after 9 weeks post-induction, many hippocampal neurons were seen with striking TUNEL reaction in the CA1 region (Figure 7C), while a cortical TUNEL reaction appeared later at 12 weeks post-induction (not shown). No colocalization of TUNEL and pH3-Ser10 (Figure 7C, red, arrows), or TUNEL and PCNA (not shown), was found. No neurons in either the non-induced mice (not shown) or any of the 8 weeks post-induction Mfn2 iKO mice showed reaction with TUNEL (Figure 7D, green), pH3-Ser10 (Figure 7D, red), or PCNA (not shown). Mfn2 iKO mice also had far fewer DAPI-positive neurons in the CA1 region (Figure 7C) compared to the CA1 region in the non-induced mice (Figure 7D), correlating with loss of NeuN staining, as shown in Figure 5.

## 4. Discussion

We recently showed how constitutive knockout of neuronal mitochondrial fusion protein Mfn2 in the cortex and CA1 region of the hippocampus in mice from birth causes eventual age-dependent neurodegeneration and cell loss [24]. In that model, Mfn2 ablation caused mitochondrial fragmentation and dysfunction, which initiates a series of events in a strict temporal order, including an early increase in oxidative stress followed by gradual inflammatory response along with loss of cytoskeletal integrity, resulting in apoptosis and eventual total neuronal loss of CA1 hippocampal neurons and some cortical neurons at later ages [24]. In the present study, we sought to examine the effects following induction of Mfn2 knockout in the adult mouse brain in an attempt to understand how Mfn2 ablation and mitochondrial fragmentation in adult neurons may result in AD-related pathological changes and neuronal loss. Consistent with our prior findings, these adult Mfn2 iKO mice also exhibited extensive neurodegeneration of hippocampal CA1, CA2, and CA3 neurons, and a variable loss of dentate gyrus neurons and cortical neurons starting at 9 weeks post-induction. The progressive neuronal loss resulted in significant loss of hippocampal and cortical volume and brain weight at the later ages post-induction. The difference in brain regions affected (CA1 in Mfn2 cKO mice in the prior study vs. CA1, 2, 3 in the Mfn2 iKO in this study) likely reflected the regional difference of CaMK-Cre expression between the two mouse models [28]. Ultrastructurally, the mitochondria in Mfn2 iKO mice were also round and swollen at 8 weeks post-induction. The mitochondria were localized in the soma, with large areas of axons devoid of mitochondria, similar to the perinuclear accumulation of mitochondria reported in PS1 mutant AD patient tissue [31]. Indeed, Mfn2, through its association with Miro, is critical for axonal transport of mitochondria in neurons [32]. At the electron microscopy (EM level, robust change in mitochondrial structure is one of the earliest changes, such that the mitochondria appear swollen and rounded and lose cristae structure, reminiscent of similar findings shown in mouse models of AD [21,33,34] as well as in human brain biopsy samples from cases of AD [4,35]. In all cases, the mitochondria become fragmented. Pathological events downstream of mitochondrial fragmentation and dysfunction also occurred in a similar temporal order, with ensuing oxidative stress preceding neuroinflammation, followed by neuronal death. These data confirmed that Mfn2 ablation in adult neurons cause mitochondrial fragmentation and dysfunction that lead to neurodegeneration through an oxidative stress response involving neuroinflammation in AD-afflicted brain regions.

While ablation of Mfn2 both constitutively or after induction in adult neurons resulted in morphological and distribution changes in mitochondria, it appeared to require a longer incubation time in the latter. In Mfn2 cKO mice, it is believed that CaMK-Cre began to express after 2–4 weeks of age, and mitochondrial abnormalities became apparent 4–6 weeks after CaMK-Cre expression. However, in the Mfn2 iKO adult mice reported in this study, mitochondrial morphological abnormalities became apparent 8 weeks after induction. 

It appears that the mitochondrial ultrastructural damage was milder, with some different features in the Mfn2 iKO mice compared to Mfn2 cKO mice: a total loss of cristae structure has commonly been found in Mfn2 cKO mice at 28 weeks of age [24]. However, a total loss of cristae is rare even in Mfn2 iKO mice 40 weeks post induction. Instead, there is striking cristae disorganization into small circular arrangements, and no normal cristae spanning the mitochondria remain in the Mfn2 iKO neurons. Similar cristae disorganization was reported in a mitochondrial myopathy [36] and human patients carrying Mfn2 mutations [37]. This cristae morphology was also elegantly reported in a yeast modeling system of mitochondria swelling by ATP and retraction with polyethylene glycol (PEG). While some normal tubular cristae were formed following the contraction, many small unattached “vesicular” cristae remained [36]. It is not clear whether such abnormal mitochondrial cristae disorganization in Mfn2 iKO mice is secondary to mitochondrial swelling or directly caused by Mfn2 ablation, however. In the latter, it is of interest to note that Mfn2 knockout could impact the processing of OPA1, a key molecule in cristae morphogenesis [38]. Given that similar mitochondrial swelling was also reported in neurons in DLP1 knockout mice [39], mitochondrial swelling appears to not be specific to Mfn2 ablation. Rather, it reflects mitochondrial dysfunction that leads to a loss of capability to maintain mitochondrial membrane permeability. We also describe other ultrastructural changes including many mitochondria with outer membrane protrusion or with internal circular cristae and compartmentalization, similar profiles to mitochondrial abnormalities reported in mitochondrial myopathy [40]. Still other mitochondria in the Mfn2 iKO neurons show loss of double outer membrane integrity, either by rupture or disintegration, morphological evidence for mitophagy [41]. Sometimes, inner membrane integrity is also lost. Similar mitochondrial swelling and membrane degeneration has been shown following ischemic cell injury [42]. Overall, these morphological deficits in the Mfn2 iKO neurons may represent intermediate steps that eventually progress to the total loss of cristae structure, as seen in the Mfn2 cKO neurons. 

Similar to Mfn2 cKO mice, significantly increased astrocyte and microglia activation in the hippocampus and cortex occurred after oxidative stress but immediately prior to neuronal loss (Figure 5). In some mice, the tissue damage was extensive, such that large hippocampal or cortical areas contained high levels of activated microglia and apoptosis yet were barren of any astrocyte immunoreactivity. This has been reported previously in other models of severe tissue damage [43]. It remains to be determined how microglia and/or astrocytes get activated in Mfn2 cKO mice. A recent study suggests that fragmented mitochondria released from microglia after inflammatory activation cause neuronal dysfunction through the activation of astrocytes [44]. A similar mechanism could be involved through the release of fragmented and dysfunctional mitochondria from Mfn2 iKO neurons.

Aberrant cell cycle-events in susceptible neurons in the AD brain have been reported by multiple groups, which is believed to mediate cell death in these neurons [45,46]. We utilized double-staining fluorescent microscopy to compare the appearance of early and late cell cycle markers with apoptosis in individual neurons undergoing degeneration in the 9 weeks and 12 weeks post-induction mice. PCNA, which is expressed early in the cell cycle from the G1/S through the G2/M phase, was found increased in both neurons and microglial cells, while pH3-Ser10, which is expressed late in the cell cycle in the G2M phase, was only found in neurons (Figure 7). While PCNA and pH3-Ser10 were often found colocalized in neurons, they never colocalized in TUNEL-positive neurons. Importantly, none of the 8 weeks post-induction mice showed any neuronal immunoreactivity for cell cycle or TUNEL. While a progression of events from oxidative stress to cell cycle re-entry to apoptosis has been reported in other models of apoptosis [47], our lack of colocalization of even late cell-cycle events and TUNEL in individual neurons suggests that death through an aberrant cell cycle may be a different form of death than apoptosis. 

In addition to mitochondrial fusion, Mfn2 is also involved in the regulation of ER–mitochondria tethering. While initial study demonstrated that Mfn2 locates to ER and interacts with Mfn1 or 2 on the mitochondrial outer membrane to mediate ER–mitochondrial tethering [48], more recent studies have suggested that Mfn2 actually plays an antagonizing role in ER–mitochondrial tethering [49,50,51]. Given the presence of different types of ER–mitochondrial tethering with different distances [52], it could be explored whether Mfn2 plays a different role in different types of ER–mitochondrial tethering. In relation to AD, Mfn2 was shown to modulate ER–mitochondria coupling through its interaction with presenilin 2 [53]. More recent studies have also demonstrated that Mfn2 is involved in the regulation of mitophagy through the Mul1–Mfn2 pathway [54]. Both ER–mitochondrial tethering and mitophagy are important for mitochondrial homeostasis, and therefore, the possibility that Mfn2 knockout could elicit neurodegeneration through abnormal ER–mitochondria tethering or mitophagy in our mouse model could not be ruled out and is an avenue that merits further investigation. 

In summary, in the current study, we demonstrated that Mfn2 ablation and mitochondrial fragmentation in adult neurons caused neurodegeneration through oxidative stress and neuroinflammation in vivo via both apoptosis and aberrant cell-cycle-dependent cell-death pathways. Maintaining normal levels of Mfn2 levels and restoration of mitochondrial dynamics may provide protection from neurodegeneration [55].
